# Bone Vascularization in Normal and Disease Conditions

**DOI:** 10.3389/fendo.2013.00106

**Published:** 2013-08-26

**Authors:** Christian Carulli, Massimo Innocenti, Maria Luisa Brandi

**Affiliations:** ^1^Department of Surgery and Translational Medicine, University of Florence, Florence, Italy

**Keywords:** bone vascular biology, bone endothelium metabolism

## Abstract

Bone vasculature is essential for many processes, such as skeletal development and growth, bone modeling and remodeling, and healing processes. Endothelium is an integral part of bone tissue, expressing a physiological paracrine function via growth factors and chemokines release, and interacting with several cellular lines. Alterations of the complex biochemical interactions between vasculature and bone cells may lead to various clinical manifestations. Two different types of pathologies result: a defect or an excess of bone vasculature or endothelium metabolism. Starting from the molecular basis of the interactions between endothelial and bone cells, the Authors present an overview of the recent acquisitions in the physiopathology of the most important clinical patterns, and the modern therapeutic strategies for their treatments.

Bone is a highly vascularized tissue, characterized by an intense turnover of neoformation and resorption. The vasculature in bone tissue is important for skeletal development and growth, modeling and remodeling, and healing processes. Endothelium is an integral part of bone tissue, and has a role in the interaction with bone cells in all the mentioned processes (1). This is based on the significative heterogeneity of endothelial cells features, as for size, tissue, and age both in physiologic (2, 3) and in pathologic conditions (4–6). Moreover, it is now clear that endothelium plays a role in the local bone metabolism, acting in a paracrine fashion on other bone stromal cells via humoral factors, such as growth factors and chemokines (1, 3). The interaction of endothelial cells and other bone cells has been interpreted, and fascinating hypotheses have been proposed over the past two decades (7–12). However, the molecular mechanisms of action that underlie this cross-talk is not yet crystal clear.

In conditions when the mechanical stability is normal and an adequate combination of cells, growth factors, and bone matrix is associated to an appropriate blood supply, important processes like bone formation, growth, and healing occur, as hypothesized according to the “diamond concept” (13). However, there are several conditions in which bone tissue loses a normal paracrine endothelium function, as happens in trauma, metabolic disorders, and genetic diseases. Also, vascular accidents may affect the integrity, affecting bone vasculature. Moreover, there are bone segments with a terminal vascularization (proximal femur, carpal scaphoid, talus) that are at high risk due to the lack of an appropriate collateral vascular network. In case of alterations in their unique blood supply, bone metabolism, and bone health are dramatically affected. More frequently, bone metabolism is inhibited, resulting in decreased bone formation.

An overview of the complex biochemical interactions between vasculature and bone cells in normal conditions and in various clinical manifestations follows.

## Endothelial and Bone Cells. The Molecular Basis of Their Interactions

Endothelial cells in bone have several functions: maintenance of vascular integrity, contribution to bone formation, and direct stimulation of osteoblasts/osteoclasts cross-talk (11, 14).

**Figure 1:**
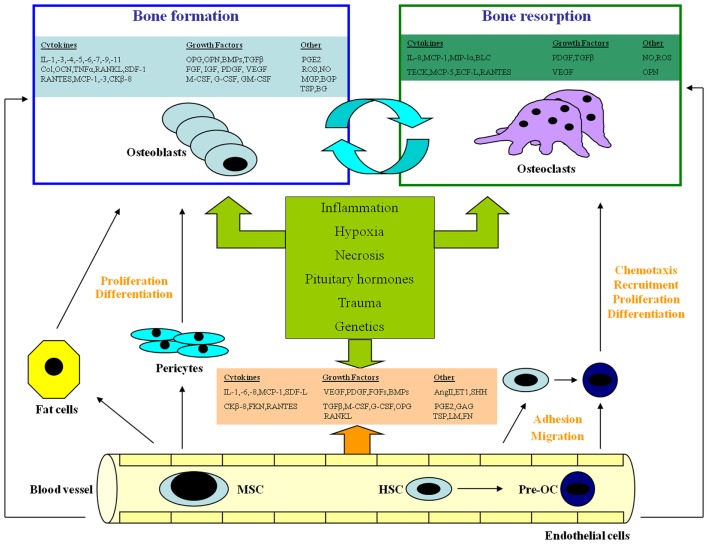
**The complex interaction among endothelial cells, osteoblasts, and osteoclasts**. EC, endothelial cell; OB, osteoblast; BMSC, bone marrow stromal cell; OC, osteoclast; pre-OC, OC precursor; TEM, transendothelial migration; MSC, mesenchymal stem cell; HSC, hematopoietic stem cell; SDF-1, stromal cell-derived growth factor-1; IL-, interleukin; MCP, monocyte chemoattractant protein; CK-8, a chemokine; FKN, fractalkine; RANTES, regulated on activation of normal T cell expressed and secreted; MIP-1, macrophage inhibitory protein-1; Col, collagen; LM, laminin; FN, fibronectin; TSP, thrombospondin; GAGs, glycosaminoglycans; VEGF, vascular endothelial growth factor; PDGF, platelet-derived growth factor; FGFs, fibroblast growth factors; OPG, osteoprotegerin; BMPs, bone morphogenetic proteins; TGF, transforming growth factor; M-CSF, macrophage-colony stimulating factor; G-CSF, granulocyte-colony stimulating factor; GM-CSF, granulocyte/macrophage-colony stimulating factor; Ang II, angiotensin-II; PGE2, prostaglandin E2; ROS, reactive oxygen species; ET-1, endothelin-1; SHH, Sonic Hedgehog; OCN, osteocalcin; OPN, osteopontin; ON, osteonectin; MGP, matrix Gla protein; BGP, bone Gla protein; BG, biglycan; IGFs, insulin-like growth factors; BLC, B-lymphocyte chemoattractant; TECK, thymus-expressed chemokine; ECF-L, eosinophil chemotactic factor-L.

In several studies carried out over the last decades, interpretations were offered to understand the molecular relationship between vasculature and bone, using both *in vivo* and *in vitro* models (Figure 1).

One key point is represented by the communication between endothelium and bone cells, based on the release of humoral factors, such as growth factors. Growth factors are critical for osteoinduction, a central process for bone repair, as occurs in fracture healing. To ensure successful bone healing, the induction of angiogenesis is needed, and marrow stromal cells (MSCs) are also used to induce bone formation (15–20). Mesenchymal elements express factors like Vascular Endothelial Growth Factor (VEGF), and it has been demonstrated that MSCs from healthy subjects are characterized by a signature profile of VEGF expression distinct from patients affected by Osteonecrosis (ON) of the hip and by Osteoarthritis (OA) (14). Both in animal models and in humans, VEGF expression was evaluated in mesenchymal cells from osteonecrotic bone with the common conclusion that VEGF enhances bone forming activity (15, 17, 19). Indeed, the expression of VEGF, assessed in human specimens from late stage ON of the femoral head, showed that osteoblasts from the reactive interface exhibited increased VEGF expression, which the investigators postulated to be a secondary phenomenon in an attempt to stimulate the ingrowth of a reparative blood supply (21). In addition, it was also found that osteoblasts derived from OA femoral heads exhibited down-regulation of VEGF after 24 h of co-incubation with glucocorticoids. Another study has suggested that there may be a strong association of a polymorphism in the VEGF genotype (-634G/C polymorphism) with ON of the femoral head in the Chinese population (22).

Prostaglandins are produced in bone as response to inflammation, injury, and mechanical load, and have been implicated in the local regulation of bone metabolism. Increased production of prostaglandins (particularly PGE2) induces in a dose-dependent fashion the overexpression of VEGF mRNA in osteoblasts in an animal model (study of VEGF mRNA expression in rat calvaria-derived osteoblast-enriched cells) (23): the result is an increase of bone turnover, as for a significant paracrine effect of VEGF, more than a supposed autocrine action (23). Support to this theory is provided also by a recent study that confirms the paracrine/intracrine function of VEGF, also able to induce differentiation both for osteoblasts and adipocytes (24). There is also another theory about how VEGF may be important in coupling bone formation and angiogenesis: some authors propose that VEGF alone is not able to promote bone regeneration in some processes, as fracture healing, but synergistically with BMPs (BMP-2, BMP-4) (25).

Some authors advanced a theory that some lines of bone stromal cells are a bone-specific type of microvascular pericytes, able to interact with the endothelium, and with high multilineage potential (26–29). This would mean that these pericytes may be considered as a reserve for any process regarding bone tissue healing (26). However, only cells near blood capillaries may give rise to bone forming cell lines (30). Indeed, bone vasculature is of crucial importance during the ossification of growth plates (31). While the growth takes place, the chondral tissue becomes thinner due to the induced apoptosis that favors substitution of the calcified cartilage with bone tissue. VEGF, the Hypoxia-Inducible Factor (HIF-1) and RANK-L are highly expressed by hypertrophic chondrocytes, to modulate local bone metabolism, angiogenesis, and osteogenesis (32–34). Moreover, VEGF expressed by the endothelium plays a role in osteogenesis via the increased production of BMPs (BMP-1, BMP-4) (35–37). In addition, factors secreted by endothelium, such as endothelin-1 and angiotensin-II, are also able to induce bone formation (35–41). The action of the endothelium may thus be considered as a coordinating function in the cross-talk between bone cells and angiogenesis. Specifically, it seems that the local release of VEGF induces both endochondral and intramembranous ossification during bone growth, bone development, bone remodeling, and bone repair (42). Similarly, osteoclasts deriving from bone marrow or circulating precursors may migrate to bone resorption sites through endothelium, which also releases cytokines able to activate the process of osteoclastogenesis (40, 43–45).

The role of the endocrine systems in the regulation of the interaction between vessels and bone is an area of great interest. Pituitary hormones such as adreno-corticotropic hormone (ACTH), thyroid-stimulating hormone (TSH), and follicle-stimulating hormone (FSH) control the synthesis of VEGF by osteoblasts (46). Particularly, ACTH induces VEGF release, TSH receptors are expressed by osteoblasts and osteoclasts, and FSH acts via TNF-α to favor bone resorption (46). Moreover, the uncontrolled VEGF release by bone marrow cells in the early stages of steroids-induced pathologic bone conditions (i.e., ON of the femoral head) may lead to vascular insufficiency related to increased endothelial gaps and hyperpermeability of the endothelial wall, resulting in inadequate bone repair (47). Functional estrogen receptors in bone endothelial cells were found in bovine models *in vitro*, suggesting a role of estrogens in bone angiogenesis and in the entire process of bone remodeling (48). Moreover, parathyroid hormone (PTH) has shown a vasodilating effect on bone vasculature (11). Also, local factors, such as the RANK/RANK-L pathway, are known to play a fundamental role in the modulation of angiogenesis and endothelial cell survival (34, 49). In addition, recent acquisitions indicate that homeobox genes are able to control the connection between angiogenesis and osteogenesis. Indeed, the Sonic Hedgehog seems to control the expression of several families of growth factors, mainly VEGFs (50). The results of this action are a direct angiogenic effect, an acceleration of the endothelial cell tube formation, and the differentiation of local mesenchymal cells in the osteogenic lineage. Finally, differential expression of genes encoding bone matrix proteins and local growth factors and chemokines has been indicated as one of the signatures in pathologic conditions, as in ON of the femoral head (51).

## Clinical Conditions of Altered Bone/Vasculature Complex

Several pathologies are the results of an altered relationship between endothelium, vasculature, and bone tissue. These are: avascular necrosis, pachydermoperiostosis, osteopetrosis, rickets, osteoporosis, inflammatory bone loss, multiple myeloma, Paget’s disease, metastatic bone disease, melorheostosis, Gorham–Stout disease (GSD), Klippel–Trénaunay syndrome (KTS), and vertebral angiomatosis (4–6, 52–79). Most of these conditions are related to a defect of vascular supply, although a few phenotypes are caused by an excessive vascularization (Table 1).

**Table 1:** **Main clinical manifestations of the altered bone/vasculature complex**.

Defect of bone vasculature	Excess of bone vasculature
Idiopathic osteonecrosis	Gohram–Stout disease
Trauma related osteonecrosis	Klippel–Trénaunay syndrome
Sickle cell anemia	Vertebral hemangioma
Thrombophilia related osteonecrosis	Pachydermoperiostosis
Bisphosphonates-related osteonecrosis of the jaws	Osteopetrosis

### Defects of the bone vasculature or endothelium metabolism

#### Idiopathic osteonecrosis

Among the diseases with a defective vasculature, the most common and studied is bone ON, also known as avascular necrosis. The main blood supply to femoral head originates from the basicervical extracapsular articular ring and ascending branch of the medial femoral circumflex artery, as well as smaller secondary contributions arising from inferior and superior gluteal arteries, and the artery of the ligamentum teres (80). A significant number of cases of ON are to be considered idiopathic. However, proximal femoral fractures with fragment displacement is the most represented extracapsular cause of vascular disruption, while intravascular embolic matters such as clots, lipids, immune complexes, or sickle cells are the most common situations of occlusion of the terminal circulation of this bone segment (81–84).

Whatever the cause of ON, the reduction of the blood supply induces a consequent decrease of the bone forming activity. Specifically, the ischemic injury induced by multiple possible causes upregulates tartrate-resistant acid phosphatase (TRAP)-positive osteoclasts. These cells typically express the TRAP protein, a glycosylated monomeric metalloenzyme, that is thought to be involved in the osteoblast differentiation, activation, and proliferation in bone resorption sites: in pathologic conditions bone cells usually begin to resorb dead trabecule of subchondral bone of the femoral head, failing day by day under repetitive weight-bearing loads related to common life activities (83, 84). ON finally consists in a collapse of the bony architecture, mostly localized at the long bone epiphysis leading to chondral damage and destruction of the articular surfaces.

Early recognition of the disorder helps prevent the progression of the disease. An important symptom is acute localized pain, and in cases of persistent pain, x-rays and MRI are extremely helpful for the diagnosis. When the disease progresses, the only way to solve the severe pain and functional limitation is a joint arthroplasty (85). Several attempts were made to understand the pathogenetic basis of bone ON. Defective bone vascularization is the most accredited hypothesis, with consequent reparative response that usually fails due to excessive bone resorption. Bone ON may be the consequence of a prolonged corticosteroid treatment. Even if to date it has not been fully clarified, many hypotheses addressing ischemic changes have been proposed. Several studies have reported a specific relationship between plasma lipoprotein(a) [Lp(a)] concentration and vascular lesions such as coronary heart disease, stroke, and carotid atherosclerosis (86). This low-density lipoprotein has a component of two disulfide-linked high molecular weight proteins, apolipoprotein(a) and apolipoprotein B100; the former is able to induce arteriosclerosis and thrombogenesis (87).

Endothelial nitric oxide synthase (eNOS) has beneficial effects on bone and vascular supply. A polymorphism in intron 4 of eNOS gene was significantly associated with idiopathic AVN in Korean patients indicating a possible protective role of nitric oxide in the pathogenesis of the disease (88, 89).

#### Osteonecrosis secondary to congenital disorders

Several congenital disorders may be complicated by ON (90). Patients older than 35 years affected by sickle cell anemia (resulting from homozygosis for the Glu6Val mutation in the hemoglobin beta chain gene-HBB) often develop an ON (91, 92). Single nucleotide polymorphisms (SNPs) in genes related to different functions (vasculature, inflammation, oxidant stress, and endothelial cell biology), and also involved in bone metabolism, have been addressed as critical points in the development of ON (90). In particular, examples are represented by: the Klotho (KL) gene (encoding a glycosyl hydrolase that participates in a negative regulatory network of the vitamin D endocrine system); a BMPs gene (BMP6), encoding for pleiotropic secreted proteins structurally related to transforming growth factor β (TGFβ) and activins, which is important for bone formation, and in association with PTH and vitamin D appears to be involved in inducing bone development by human bone marrow-derived mesenchymal stem cells; and the Annexin-2 (ANXA2) gene (encoding for a member of the calcium-dependent phospholipid binding protein family and regulating the cell growth) (90).

Primary thrombophilia and hyperfibrinolysis appear to be common, heritable risk factors for bone ON by leading to an intravascular coagulation. Particularly, heterozygosity for the thrombophilic Leiden mutation of the factor V gene is considered a risk factor for ON of the jaws. Furthermore, alterations in the expression of this gene associated with a treatment with exogenous estrogens have been addressed as the causes of hip ON (93). Recently, alterations of the factor V Leiden and the prothrombin 20210A gene mutations have been associated with a higher incidence of ON of the knee (94).

#### Bisphosphonate-related osteonecrosis of the jaws

Among secondary ON, BRONJ is a debated clinical condition arising in case reports during the last decade, characterized by a heterogeneous pattern of alterations including ulceration of the oral mucosa, ON, and deep infection of the mandible and/or maxilla persisting for more than 8 weeks (95, 96). A specific risk factor was considered an oral surgical procedure in patients affected by tumoral conditions treated by long-term endovenous administration of high doses of aminobisphosphonates (97).

Even if its incidence to date is considered very low (approximately 0.01% for oral administration; 0.8–12% for intravenous injection) (98), the fact that treatment with bisphosphonates in the prevention of fragility fractures is very diffused has caused reasonable concern and justified particular attention. The role of bisphosphonates on this pathology has yet to be documented, given the lack of evidence in humans, and the recent evidence that large animals (dogs) treated by high doses of bisphosphonates, corticosteroids, or both have demonstrated necrotic or exposed bone after dental extraction, as shown in rats (99, 100).

Three bisphosphonates-induced mechanisms have been proposed in BRONJ: remodeling suppression, disrupted angiogenesis, and infection (99). Despite the fact that there are no data of the effects of bisphosphonates on jaw bone turnover in humans, information in animal models indicates a low remodeling in the jaws in terms of intracortical metabolism after bisphosphonates administration (101, 102). The presence of “non-viable osteocytes” and zones of matrix necrosis observed in a population of dogs after administration of high intravenous doses of zoledronate is another demonstration of intracortical bone remodeling suppression (103). Regarding the effects of bisphosphonates on angiogenesis, there is no evidence that the necrotic regions have a reduced vascular supply. However, high doses of bisphosphonates have been demonstrated to significantly suppress the vessel sprouting in cultured tissue chambers implanted subcutaneously in mice, and the vessel density in rats and humans (104, 105). This is probably related to a slower activity of the new remodeling units and their related vessels induced by the remodeling action of bisphosphonates. Finally, bisphosphonates have been shown to inhibit T-lymphocyte activation and proliferation *in vitro*, and to suppress the production of several pro-inflammatory cytokines by both lymphocytes and monocytes (particularly Il-1β, IL-6, TNFα) (106–109). This may explain the insidious forms of infection (related to the universal presence of *Actinomyces*) associated to a significant amount of cases of BRONJ (106, 107). A recent discovery regards the effect of BFs (alone or in combination with cortisonics) in the direct toxicity of animal oral mucosa by higher levels of apoptosis and lower levels of MMP-9 in the epithelial (96, 110).

### Excesses of bone vasculature or endothelium metabolism

In another series of clinical manifestations, pathogenesis is related to redundant alterations in the bone vascular supply, such as in GSD, KTS, and vertebral hemangioma.

#### Gorham–Stout disease

Also called “phantom bone,” “disappearing or vanishing bone disease,” “hemangiomatosis,” and “lymphangiomatosis,” GSD belongs to the family of the “cystic angiomatosis,” i.e., severe pathologic conditions characterized by disseminated multifocal vascular lesions of the skeleton with possible visceral involvement (111, 112). GSD was described as a condition involving bones (mainly the humerus, pelvic girdle, and skull), more frequent in men than women, with local an aggressive tendency, a rare self-healing behavior, and related to a marked proliferation of thin-walled capillaries without clear features of ON. Often associated to trauma (113), it is usually discovered after a pathologic fracture. The most important feature is a hyperemia with subsequent excess of bone destruction and osteoclastic activity (probably due to elevated serum levels of IL-6, IL-1, and TNF) with respect to bone formation (82, 114).

In our recent experience, a significant increase of serum Osteopontin (OPG) and Osteoprotegerin (OPG), both of which are bone matrix proteins acting as markers of a bone metabolism, has been demonstrated in patients affected by GSD as a probable incomplete compensatory self-defense mechanism (115). OPG is able to capture the RANK-L, inhibiting the differentiation and activation of osteoclasts, and it is also expressed by endothelial cells acting as antiapoptotic factor: high levels of OPG and OPN may reflect an attempt of self-defense by endothelium after aspecific bone damage (1, 116). In late stages, hypervascular fibrosis substitutes the zones of bone resorption.

Gorham–Stout disease related to congenital disorders is a diagnosis of exclusion, after ruling out other differential diagnoses (neoplasms, infections, and metabolic or endocrine disorders, idiopathic osteolysis) (115). The osteolytic process may be painless, allowing the patient to continue full activity while bone destruction occurs, making the patient susceptible to pathological fractures in the affected bones.

#### Klippel–Trénaunay syndrome

Klippel–Trénaunay syndrome is a congenital malformation with a low incidence (<1:10000; similar in males and females). It is characterized by mixed vascular (capillary and venous) malformations associated with abnormal growth in the extremities, muscle hypoplasia or hypotrophy, and intramuscular lymphatic lesions. KTS is the most representative example of combined vascular malformation: histologically, a triad of capillary malformation, atypical varicose veins (also known as marginal or anomalous lateral veins) or venous malformations, and hypertrophy of soft tissues and/or bone, is very frequent (117).

Its origin is still debated, probably correlated to mutations of genes encoding for angiogenic factors, such as VG5Q (AGGF1 – angiogenic factor with G patch and FHA domains-1) and RASA1 (Ras p21 protein activator 1), both located in chromosome 5 (118, 119). Clinically, there are two types of KTS: simple and complex. Simple KTS has a blotchy/segmental port-wine stain (PWS) and a better prognosis. Complex KTS features geographic PWSs, often includes deep venous system aplasia or hypoplasia, and has a higher risk of lymphatic involvement and a greater number of complications.

#### Vertebral hemangioma

Until MRI was made available worldwide, vertebral hemangiomas were almost unknown. Given the high sensibility and specificity of MRI for fat and vascular tissues, these lesions were detected mostly as “incidentaloma,” the vertebral hemangioma being a dysembryogenetic (hamartomatous) mass, composed of thin-walled vessels lined by flat, bland endothelial cells infiltrating the medullary cavity between bone trabecule (120–123).

Very common, and frequently multiple, the prevalence of hemangiomas seems to increase with age and is greatest after middle age, with a slight female predilection. Most hemangiomas are seen in the thoracic and lumbar spine. They are usually confined to the vertebral body, although they may occasionally extend into the posterior elements. Most spinal hemangiomas are asymptomatic (124). Occasionally, vertebral hemangiomas may increase in size and compress the spinal cord and nerve roots. Compressive vertebral hemangiomas can occur in patients of any age, with a peak prevalence in young adults, preferentially occurring in the thoracic spine (52, 122, 124).

## State of the Art of the Therapeutic Approaches, and the Future Horizons

Whatever the type of alteration in the bone vascular supply and metabolism, antiresorptive drugs are considered to date the elective treatment (15, 103, 104, 109, 119, 125–130). Bisphosphonates may allow the reduction of bone loss and resorption in high turnover conditions, acting also for their strong antiangiogenic activity in hypervascularization. On the other hand, bisphosphonates may also operate to prevent or limit bone resorption secondary to a down-regulate local vascular or bone metabolism (i.e., ON), by inhibiting the osteoclasts action in favor of the osteoblastic activity. Over the decades, several types of bisphosphonates have been proposed, and different forms of administration have been tested. In addition, new drugs have been studied and introduced in specific conditions, with extension of the indications.

Osteonectin of the femoral head is one of the best known pathologies, and its treatment depends on the stage at the moment of the diagnosis. The choice of treatment is independent frp, the main cause of ON, either idiopathic or secondary. Early stages may be treated by a combination of limited weight bearing on the affected side (use of crutches), activity modification, bisphosphonates administration (preferably via a parenteral route), and physical therapy (magnetic fields, hyperbaric therapy) (131, 132). Medium-stages ON may be treated by a well-known surgical procedure, core decompression of the femoral head and neck, in association with several elements that over the decades have been proposed: bone grafting, acrylic cement, vascularized bone (fibular) grafts (126, 133–135). However, the best combination now seems to be filling with a biological composite made of bone graft enriched with a concentrate of autologous bone marrow cells derived from an iliac crest harvest and a bioceramic, to ensure a biomechanical support (85). Ever since the preliminary reports, this technique has shown, both *in vitro* and *in vivo*, hystomorphometric, radiologic, and clinical success (136–138). Late stages of ON may only be treated by a total hip replacement, given the severe articular involvement.

New trends in the medical and pre-prosthetic surgical treatment of ON have been proposed. Autologous adipose-derived stem cells have been employed in rabbit models for the treatment of the steroid and avascular induced ON of the femoral head obtaining a bone response with increased trabecular density and volume coupled with intense neoangiogenic phenomena (139). Encouraging results were obtained by the use of tetramethylpyrazine, a small molecule able to bind VEGF to its receptor blocking its signaling pathway in the treatment of the steroid-induced ON, as local intraosseous injections of discrete doses of these experimental drugs have demonstrated efficacy in patients at risk of bone collapse (47, 140).

As reported, bisphosphonates also represent the treatment of choice of the redundant alterations of bone vasculature (particularly GSD), given their ability to inhibit vascular proliferation and induce endothelial cell apoptosis (104, 127, 129, 130, 141). Bisphosphonates effectiveness in GSD may be demonstrated by clinical and radiologic assessment of the patients, and also by laboratory study of the serum levels of bone metabolism markers. Pamidronate given by intravenous infusion showed a dramatic decrease of serum OPN and IL-6. Future challenges will regard the possible indication of Denosumab, a monoclonal antibody acting as a potent antiresorptive drug recently registered for the prevention of fragility fractures (142) in the treatment of GSD (115). As reported in literature, GSD and other forms of angiomatosis may need a multidisciplinary approach, consisting in medical therapies (bisphosphonate and interferon administration), radiation therapy, and surgery (bone grafting with biological composites enriched by MSCs or prostheses) (125, 128, 143, 144).

Rare conditions, such as KTS, have been treated with several approaches, even if it is clear that a multidisciplinary involvement is necessary in order to manage the different alterations. A conservative treatment is usually proposed for dermal lesions, and to manage any vascular or osteoporotic risk. A combination of physical therapy, compressive bandages, stockings, low molecular weight heparin, bisphosphonates, and anabolics (i.e., Teriparatide and Strontium Ranelate) have also been adapted. A surgical approach is indicated in cases of severe vascular or dermal damage or orthopedic complications (117, 119).

Vertebral hemangiomas are to date considered frequent benign alterations of the dorsal and lumbar spine: generally no specific treatment is proposed. However, a hemangioma may cause radicular pain and peripheral neurologic impairment; a careful differential diagnosis is essential to exclude any doubt of a malignant condition, such as severe osteoporosis (120, 123). Standard medical management is addressed in order to solve the symptoms. Rarely, a significant compression on nerve roots may need surgery, generally with conventional procedures (vertebroplasty, kyphoplasty) (145).

Finally, gene therapies have recently been studied to up- or down-regulate the bone vasculature by bioactive molecules in specific pathologic situations with promising outcomes (146). The delivering of a “suicide gene” able to selectively eliminate specific cells involved in neoplastic tissue vasculature could induce a substantial inhibition of angiogenesis without systemic toxicity (147).

Even if progress in understanding the metabolic regulation of bone vasculature has been made during the last decades, much more has to be understood about the actual communication between bone vessels and their components and bone cells. Animal models helped us to begin the comprehension of this dense signaling network, but the point in question is to correlate the molecular interactions with the various clinical patterns that nowadays affect patients. In some cases, as for ON of femoral head or GSD, we are now trying to apply in selected patients what we have learned from laboratory, but these crucial steps have to be made carefully for all other pathologies.

The above mentioned pharmacological and molecular agents represent promising interventions of the present and future, but for some time still, efforts will have to be made to enlarge the therapeutic armamentarium of the clinicians.

## Conflict of Interest Statement

The authors declare that the research was conducted in the absence of any commercial or financial relationships that could be construed as a potential conflict of interest.
